# Self-Medication Practices among Community of Harar City and Its Surroundings, Eastern Ethiopia

**DOI:** 10.1155/2018/2757108

**Published:** 2018-07-25

**Authors:** Sara Mamo, Yohanes Ayele, Mesay Dechasa

**Affiliations:** ^1^Wollo Tertiary Care and Teaching Hospital, Wollo, Ethiopia; ^2^Department of Clinical Pharmacy, School of Pharmacy, College of Health and Medical Sciences, Haramaya University, Harar, Ethiopia

## Abstract

**Purpose:**

Self-medication practice is often associated with irrational medication use. The aim of this study was to assess self-medication practices among community of Harar City and its surroundings, Eastern Ethiopia.

**Methods:**

A cross-sectional study was conducted through exit interview in selected drug outlets of Harar City among 370 clients from March to April, 2017. The data was coded and entered into epi-data and processed and analyzed using SPSS version 20.

**Results:**

Many participants practiced self-medication to alleviate their headache (30.30%), to treat their respiratory disorders (29.50%), and to treat their gastrointestinal disorders (27%). More than half (57.8%) of study participants declared that they were practicing self-medication due to prior experience and seeking less expensive service (20.50%). Two-fifths of them (40.3%) reported pharmacy professionals as source of information while 18.9% of respondents were advised by neighbors, friends, or relatives. About one-third (31.9%) of them did not have any source of information for self-medication practice. The most common type of drug used for self-medication by the participants was analgesic (42.2%). Approximately one-third (31.1%) of the subjects were expecting to be counseled by the pharmacy professionals about the drug side effects and to be helped in selecting their self-medication drug (30.3%).

**Conclusion:**

Varieties of medications were used among study participants ranging from antipain to that of antibiotics for different complaints including headache, respiratory complaints, and gastrointestinal problems. Experience with drugs and diseases as well as affordability were frequently reported reasons for self-medication practice. Participants had different views toward the role of pharmacy professionals. Hence, it is very important to educate patients on responsible use of medications and create awareness on the role of pharmacist in self-selected medication use in community.

## 1. Introduction

According to World Health Organization (WHO), self-medication is an element of self-care based on selection and use of medicines by individuals to treat self-recognized illnesses or symptoms [1]. Self-medication is practiced for quick and effective relief of symptoms of minor ailments without medical consultations to reduce burden on health care services, most especially in understaffed, inaccessible remote areas [2, 3].

Self-medication has a positive impact on individuals and health care systems if practiced correctly. It allows patients to take responsibility, build confidence to manage their own health, and save time spent in waiting for a doctor, and it may help to decrease health care costs [1, 4, 5]. Nevertheless, self-medication practice is highly prone to inappropriate use and has its own drawbacks resulting in wastage of resources, increased resistance of pathogens, and increased adverse reaction [6, 7]. Self-medication practices can also lead to incorrect self-diagnosis, delays in seeking appropriate care, dangerous drug interactions, incorrect dosage, incorrect choice of medication, and risk of dependence and drug abuse [2, 8].

Study conducted in different setting indicates high prevalence of self-medication practice [9–11]. In order to reduce irrational medication use due to self-medication, community pharmacy professionals can play key role through providing support and advice about medicines to the general public [12–15]. However, studies showed poor understanding of public on the role of community pharmacist in self-care [16, 17].

In developing countries like Ethiopia, people are using not only nonprescription drugs but also prescription drugs without supervision [18]. However, there is no study done in Harar City, Eastern Ethiopia, on the current status of self-medication practices and communities' view toward the role of community pharmacy professionals. Hence, this study aimed to assess self-medication practices and public perspectives toward role of community pharmacy professionals in Harar City.

## 2. Materials and Methods

### 2.1. Study Area and Period

This study was conducted among community of Harar City and its surroundings from March to April, 2017. Harar, the capital city of Harari regional state, is located in the Eastern part of Ethiopia, at a distance of 525 Km from Addis Ababa, the capital city of Ethiopia. During the study period there were 16 pharmacies and 44 drug stores in the Harar City.

### 2.2. Study Design and Study Population

A cross-sectional study was conducted among clients who visited drug outlets during study period. All residents of Harar City and its surroundings were the source populations and all clients who visited selected private drug retail outlets for self-medication within the study period were the study populations. Participants aged 18+ who purchased medication without prescription were included in the study. Clients who presented to collect drugs for other clients were excluded from the study.

### 2.3. Sample Size and Sampling Technique

The actual sample size for the study was determined using the formula for single population proportion by assuming 5% marginal error, 95% confidence interval,* ∂* (alpha) = 0.05, and 50% of the prevalence rate of self-medication to get total sample size of 384. Stratified random sampling technique was used to select representative drug retail outlet. For each selected drug retail outlets, number of the participants to be interviewed was allocated based on patient flow to the selected outlets.

### 2.4. Data Collection and Analysis

A structured questionnaire was used for data collection. The pretest was conducted to check the functionality of the tools. The training was given for data collectors on the content of data collection tools and ways of approaching the interviewee. After the data collection, the questionnaires were checked for its completeness and consistency. Finally, it was coded and entered into epi-data, processed, and analyzed using SPSS version 20. The findings were presented by frequencies, percentages, and summary measures and displayed using tables.

### 2.5. Ethical Consideration

The study protocol was approved by the school of pharmacy research ethics review committee, Haramaya University. A formal permission letter was obtained from College of Health and Medical Sciences and submitted to drug retail outlets managers and the purpose of the research and how its premise is selected were also communicated. Before the data collection, verbal consent was obtained from each participant by informing them of the purpose of the study.

### 2.6. Operational Definitions

*Pharmacy Professionals*. Pharmacy practitioners who were working in drug outlets regardless of their educational level.

*Drug Outlets. *Refers to both community pharmacies and drug stores.

## 3. Results

### 3.1. Sociodemographic Characteristics of Participants

A total of 370 clients visiting drug outlets in Harar City participated in the survey while further 14 refused to participate, giving a response rate of 96.4%. The mean age of respondents was of 32 with a standard deviation of 11.15 years. Proportion of females was 53% as compared to males. More than half (54.9%) of respondents were married. Regarding economic status, the mean and standard deviation of respondents monthly income was 3179 and 2643.66, ETB, respectively.  Table 1 shows sociodemographic characteristics of participants.

### 3.2. Self-Medication Practice of Participants

As it is indicated in Table 2, about one-third (30.3%) of the subjects used self-medication to alleviate their headache, to treat their respiratory disorders (29.5%), and to treat their gastrointestinal disorders (27%). When asked about the reason for self-medication, more than half (57.8%) respondents mentioned their previous experience while 20.50% of them practiced self-medication seeking less expensive drug or service. Regarding their source of the information, on self-medication, two-fifths of them (40.3%) reported pharmacy professionals as source of information while 18.9% of respondents were advised by neighbors, friends, or relatives. About one-third (31.9%) of them did not have any source of information for self-medication practice. In this study, analgesic was the most commonly (42.2%) used medicine followed by respiratory drugs (31.1%) and gastrointestinal drugs (19.50%).

### 3.3. Participants' Ways of Request for Self-Medication

As to patients approach to requesting a self-medication, about half of the subjects collected the medication by telling professionals the name of the drug while 43% of them asked professionals by telling them their symptoms. Participants were also quizzed about their expectation from pharmacy professionals. Just under one-third of respondents (31.1%) expected to be counseled by the pharmacy professionals about the drug side effects and how to avoid them and 30.3% of them were expecting help in the drug selection process (Table 3).

## 4. Discussion

The primary objective of this study was to assess self-medication practices among community of Harar City. In this study, the common chief compliant was headache and respiratory and gastrointestinal disorders. The major deriving reason for self-medication was previous experience of drugs and disease and cheapness of such practice compared to visiting health care setting such as clinic and hospitals. Pharmacy professionals followed by neighbors, friends, or relatives were the main source of information for self-medication practice. In this study, analgesic was the most commonly used medicine followed by respiratory drugs and gastrointestinal drugs.

In this study, headache compliant was leading (30.3%) cause for self-medication followed by respiratory (29.5%) and gastrointestinal disorders (27%). A systematic review conducted in Ethiopia reported fever/headache, gastrointestinal tract diseases, and respiratory diseases as the commonest illnesses for which self-medication was taken, accounting for an average of 30.5%, 19.7%, and 18.3% of self-medication use, respectively [18]. This finding is also consistent with studies done elsewhere [19–21]. For the aforementioned complaints self-medications seem more logical than visiting doctors because often the pharmacy is most easily accessible and more time-saving. Although those symptoms seem less severe and manageable, pharmacy professionals and patients have to be familiar with alarming signs and symptoms that require physicians' visit. In addition, pharmacy professionals should reassure clients without recommending drugs as not all illness require medication.

In our study, majority of study subjects (57.8%) declared that they were practicing self-medication since they have had prior experience while 20.5% of them practiced self-medication because of its affordability. Mildness of the ailments was also frequently stated as a reason. This result is in agreement with study done elsewhere [22–24]. In spite of cheapness of self-medication practice, there might be long term underlining health damage for certain medical cases if they are not managed timely and properly. Hence pharmacy professional should be familiar with such medical cases and should alert clients regarding appropriate actions.

In the present study, the most common sources of information were pharmacy professionals (40.3%) and self-decision without any information (31.9%), followed by neighbors, friends, or relatives (18.9%) and from reading drug related materials (8.1%) such as labels, leaflets, or promotion about the medication. A Study done in Jimma town showed that about 48.02% of self-medicated individual's source of information was reported from personal drug outlets [22] which is almost similar to this study. However, study conducted in Mekele reported common information sources for self-medication as self-decision (64.00%), friends (31.25%), and media and reading material (14.10%), while the least common information source for self-medication was pharmacy professionals or druggists (9.40%) which is in contrast with this study [25]. This disagreement might be due to the difference in study population as a later study was conducted among university students. It is understandable that the users get information for self-medication from different sources. However, the community should be aware of the fact that not all sources of information are genuine and be able to prove the truthfulness of information.

Regarding category of drugs used for self-medication, in current study, analgesics were most commonly used drugs (42.2%), followed by drugs taken for respiratory problem (31.10%) and gastrointestinal drugs (19.50%). This finding is in line with studies conducted elsewhere [17, 20]. However, the major difference was seen on report regarding antimicrobial use. In our study, only 3% of participants reported antimicrobial use. This might be due to participants' lack of understanding about type of drugs they used. Although fewer participants reported use of antibiotics, it is very important to discourage self-medication with antibiotics.

Public is expected to trust and benefit from expertise of community pharmacy professionals on medication related issues. Nevertheless, in this study, only 31.1% of participants were expecting consultation from pharmacy professionals about the drug side effects and only 30.3% of them were seeking help in selection of self-medication drug which is relatively low compared to study done elsewhere [9, 26]. This disparity may be due difference in study area which shows lack of awareness on role of community pharmacy professionals in this study area. Community pharmacy professionals can play significant role in primary care because of their easy access to the community. However, these professionals are often underutilized due to different reasons; one of the reasons being community lack of awareness about potential roles and mistrust. Hence, it is very important to create awareness among community about the roles and capabilities of community pharmacy personnel regarding the medication related issues.

## 5. Conclusions

In present study, headache, respiratory complaints, and gastrointestinal problems were common reasons for which patient sought self-medication. The practitioners also mentioned their previous experience and affordability of the drug in pharmacy as deriving factors. Although many self-medication users had information about the drug from pharmacist, significant number of subjects had no knowledge about the medication they used. Antipain medications were the most commonly used medicine followed by respiratory drugs and gastrointestinal drugs. In current study, requesting the medicine by telling professionals the name of the drug was common followed by asking professionals by telling them their symptoms. Furthermore, participants had different views toward the role of pharmacy professionals.

## Figures and Tables

**Table 1:** Sociodemographic characteristics of participants in Harar City and its surroundings from March to April, 2017(n=370).

**Variable**	**Frequency **	**Percentage (%)**
**Age (years)**		
** **18-25	123	33.2
** **26-33	128	34.6
** **34 and above	119	32.2
Mean (SD)	32(11.15)	
**Gender**		
** **Female	196	53.0
** **Male	174	47.0
**Ethnicity**		
** **Oromo	126	34.1
** **Amhara	118	31.9
** **Hader	56	15.1
** **Tigre	28	7.6
** **Somali	16	4.3
** **Others	26	7
**Religion**		
** **Orthodox	175	47.3
** **Muslim	154	41.6
** **Protestant	34	9.2
** **Others	7	1.9
**Educational **		
** **Illiterate	27	7.3
** **Read and write	10	2.7
** **Primary school	39	10.5
** **Secondary school	94	25.4
** **College and above	200	54.1
**Marital status**		
** **Single	136	36.8
** **Married	203	54.9
** **Divorced	18	4.8
** **Widowed	13	3.5
**Occupations **		
** **Merchant	145	39.2
** **Employee	107	28.9
** **Daily labor	37	10
** **House wife	63	17
** **Others	18	4.9
**Monthly income**		
** **Nondisclosed	175	47.3
** **400-2000	78	21.1
** **2001-3600	63	17.0
** **3601-5200	12	3.2
** **>5201	42	11.4
** **Mean (SD)	3179(2643.66) Birr	

*∗* ETB, Ethiopian Birr, $1=28ETB, SD: standard deviation.

**Table 2:** Self-medication practice among participants in Harar City and its surroundings from March to April, 2017(n=370).

**Variables**	**Responses**	**Frequency **	**Percentage (%)**
Major chief complaints			
	Headache	112	30.3
	Respiratory disorders	109	29.5
	Gastrointestinal disorders	100	27.0
	Fever	26	7.0
	Skin disorders	23	6.2
Reason for self-medication			
	Less expensive	76	20.5
	Prior experience of drug or disease	214	57.8
	Disease is not serious	49	13.2
	Emergency care	30	8.1
	Others*∗*	1	0.3
Sources of information			
	Pharmacist	149	40.3
	No information	118	31.9
	Advised by neighbors, friends, or relatives	70	18.9
	Labels and leaflets or promotional materials	30	8.1
	Others∧	3	0.8
Medications used			
	Analgesic / antipyretics	156	42.2
	Respiratory drugs	115	31.1
	GI drugs	72	19.5
	Oral rehydration salt	2	0.5
	Antimicrobials	11	3.0
	Others*∗∗*	14	3.8
**Adverse drug reaction**			
	Yes	44	11.9
	No	326	88.1

*∗* indicates less waiting time; ∧ indicates other professionals, past experiences; *∗∗* indicates vitamins and anthelmintics.

**Table 3:** Participants' ways of request for self-medication in Harar city and its surroundings from March to April, 2017(n=370).

**Variables**	**Responses**	**Frequency**	**Percentage (%)**
**Self-medication request**			
	By telling professionals the symptom of illness	159	43
	By telling professionals the name of the drug	183	49.5
	By showing an old sample/package of the drugs	16	4.3
	By describing the physical characteristics of the drugs	12	3.2
**Participants expectation from pharmacy professionals**			
	To counsel them about drug side effects and how to avoid it	115	31.1
	To help them in selecting drugs	112	30.3
	To counsel them about diseases	76	20.5
	To monitor their health's progress to ensure the safety and effectiveness of drugs	35	9.5
	To label their drugs for self-medication	32	8.6

## Data Availability

The data used to support the findings of this study are available from the corresponding author upon request.

## Additional Points

*Limitations of the Study*. We were not able to establish cause and effect relationship since we included only participants who were in drug outlets for self-medication in the study. The present study had also limitations regarding its generalizability as it was not conducted in multicenter.
